# The McKenzie method for the management of acute non-specific low back pain: design of a randomised controlled trial [ACTRN012605000032651]

**DOI:** 10.1186/1471-2474-6-50

**Published:** 2005-10-13

**Authors:** Luciana AC Machado, Chris G Maher, Rob D Herbert, Helen Clare, James McAuley

**Affiliations:** 1 Back Pain Research Group, School of Physiotherapy, The University of Sydney, PO Box 170, Lidcombe, NSW, 1825, Australia; 2 Private Practice, 16 Ayres Road, St Ives, NSW, 2075, Australia

## Abstract

**Background:**

Low back pain (LBP) is a major health problem. Effective treatment of acute LBP is important because it prevents patients from developing chronic LBP, the stage of LBP that requires costly and more complex treatment.

Physiotherapists commonly use a system of diagnosis and exercise prescription called the McKenzie Method to manage patients with LBP. However, there is insufficient evidence to support the use of the McKenzie Method for these patients. We have designed a randomised controlled trial to evaluate whether the addition of the McKenzie Method to general practitioner care results in better outcomes than general practitioner care alone for patients with acute LBP.

**Methods/design:**

This paper describes the protocol for a trial examining the effects of the McKenzie Method in the treatment of acute non-specific LBP. One hundred and forty eight participants who present to general medical practitioners with a new episode of acute non-specific LBP will be randomised to receive general practitioner care or general practitioner care plus a program of care based on the McKenzie Method. The primary outcomes are average pain during week 1, pain at week 1 and 3 and global perceived effect at week 3.

**Discussion:**

This trial will provide the first rigorous test of the effectiveness of the McKenzie Method for acute non-specific LBP.

## Background

In Australia, low back pain (LBP) is the most frequently seen musculoskeletal condition in general practice and the seventh most frequent reason for consulting a physician[1,2]. According to the Australian National Health Survey, 21% of Australians reported back pain in 2001; additionally, the Australian Bureau of Statistic's 1998 Survey of Disability, Ageing and Carers estimated that over one million Australians suffer from some form of disability associated with back problems[1].

LBP poses an enormous economic burden to society in countries such as the USA, UK and The Netherlands[3]. In the largest state in Australia, New South Wales, back injuries account for 30% of the cost of workplace injuries, with a gross incurred cost of $229 million in 2002/03[4]. It is expected that most people with an acute episode of LBP will improve rapidly, but a proportion of patients will develop persistent lower levels of pain and disability[5,6]. Those patients with chronic complaints are responsible for most of the costs[6]. Effective treatment of acute LBP is important because it prevents patients from developing chronic LBP, the stage of LBP that requires costly and more complex treatment.

There is a growing concern about effectiveness of treatments for LBP, as reflected in the large number of systematic reviews published in the last 5 years addressing this issue. [7-12]. Despite the large amount of evidence regarding LBP management, a definitive conclusion on which is the most appropriate intervention is not yet available. A comparison of 11 international clinical practice guidelines for the management of LBP showed that the provision of advice and information, together with analgesics and NSAIDs, is the approach consistently recommended for patients with an acute episode[13]. Most guidelines do not recommend specific exercises for acute LBP because trials to date have concluded that it is not more effective than other active treatments, or than inactive or placebo treatments[8]. However, some authors have suggested that the negative results observed in trials of exercises are a consequence of applying the same exercise therapy to heterogeneous groups of patients. [14-16]. This hypothesis has some support from a recent high-quality randomised trial in which treatment based on a diagnostic classification system led to larger reductions in disability and promoted faster return to work in patients with acute LBP than the therapy recommended by the clinical guidelines[17].

In 1981, McKenzie proposed a classification system and a classification-based treatment for LBP labelled Mechanical Diagnosis and Treatment (MDT), or simply McKenzie Method[18]. Of the large number of classification schemes developed in the last 20 years [19-26], the McKenzie Method has the greatest empirical support (*e.g*. validity, reliability and generalisability) among the systems based on clinical features[27] and therefore seems to be the most promising classification system for implementation in clinical practice.

Physiotherapists commonly adopt the McKenzie Method for treating patients with LBP[28,29]. A survey of 293 physiotherapists in 1994 found that 85% of them perceived the McKenzie Method as moderately to very effective[28]. Nevertheless, a recent systematic review concluded that there is insufficient evidence to evaluate the effectiveness of the McKenzie Method for patients with LBP [30]. A critical concern is that most trials to date have not implemented the McKenzie Method appropriately. The most common flaw is that all trial participants are given the same intervention regardless of classification, an approach contradictory to the principles of McKenzie therapy.

The primary aim of this trial is to evaluate whether the addition of the McKenzie Method to general practitioner (GP) care results in better outcomes than GP care alone for patients with acute non-specific LBP when effect is measured in terms pain, disability, global perceived effect, and persistent symptoms.

## Methods

The University of Sydney Human Research Ethics Committee granted approval for this study.

### Study sample

One hundred and forty eight participants with a new episode of acute non-specific LBP who present to GPs will be recruited for the study. A new episode of LBP will be defined as an episode of pain lasting longer than 24 hours, preceded by a period of at least one month without LBP and in which the patient did not consult a health care practitioner[31]. Participants will be screened for eligibility at their first appointment with the GP according to the inclusion and exclusion criteria.

#### Inclusion criteria

To be eligible for inclusion, participants must have pain extending in an area between the twelfth rib and buttock crease (this may or may not be accompanied by leg pain); pain of at least 24 hours duration; pain of less than 6 weeks duration; and they need to be eligible for referral to private physiotherapy practice within 48 hours.

#### Exclusion criteria

Participants will be excluded if they have one of the following conditions: nerve root compromise (defined as 2 positive tests out of sensation, power and reflexes for the same spinal nerve root); known or suspected serious spinal pathology; spinal surgery within the preceding 6 months; pregnancy; severe cardiovascular or metabolic disease; or inability to read and understand English.

Recruiting GPs will record the number of patients who are invited to participate, the number who decline to participate, and the number of screened patients who are ineligible and their reasons for declining participation or ineligibility. Written consent will be obtained for each participant.

**Figure 1:**
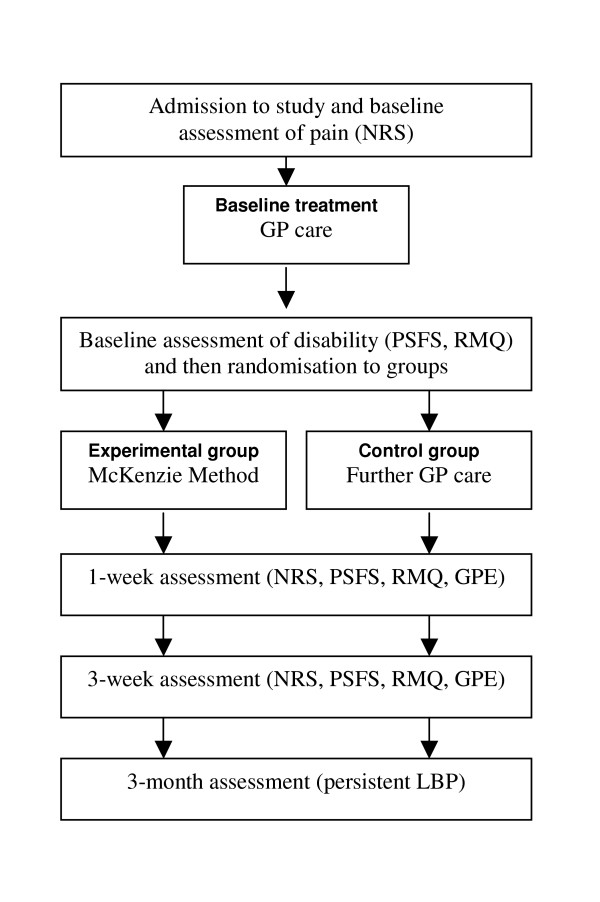
**Flow of participants through the study. **Legend: GP – General practitioner; NRS – Numeric pain rating scale; PSFS – Patient-specific functional scale; RMQ – Roland-Morris questionnaire; GPE – Global perceived effect; LBP – Low back pain.

Subjects who volunteer to participate and satisfy the eligibility criteria will receive baseline treatment and then be randomly allocated to one of the study groups. To ensure equal-sized treatment groups, random permuted blocks of 4–8 participants will be used[32]. Randomisation will be stratified by Workcover compensation status. The stratified random allocation schedule will be generated by a person not otherwise involved in recruitment, assessment or treatment of subjects and the randomisation sequence will be placed in sequentially numbered, sealed envelopes. The flow of participants through the study is detailed in Figure 1.

### Outcome measures

The McKenzie protocol is thought to promote rapid symptom improvement in patients with LBP[33,34] and this is one of the reasons that therapists choose this therapy. Therefore it is important to focus assessment on short-term outcomes. The primary outcomes will be:

1. Usual pain intensity over last 24 hours recorded each morning in a pain diary over the first week. Pain will be measured on a 0–10 numerical rating scale (NRS). The unit of analysis will be the mean of the 7 measures[35];

2. Usual pain intensity over last 24 hours (0–10 NRS) recorded at 1 and 3 weeks[35];

3. Global perceived effect (0–10 GPE) recorded at 3 weeks.

The secondary outcomes will be:

1. Global perceived effect (0–10 GPE) recorded at 1 week;

2. Patient-generated measure of disability (Patient-Specific Functional Scale; PSFS) recorded at 1 and 3 weeks[36];

3. Condition-specific measure of disability (Roland Morris Questionnaire; RMQ) recorded at 1 and 3 weeks[37];

4. Number of patients reporting persistent back pain at 3 months.

Following the screening consultation in which the inclusion and exclusion criteria are assessed, the GP will supervise the baseline measurement of pain. All patients will then receive an assessment booklet and a pre-paid envelope in which all other self-assessed outcome measures are to be recorded and sealed. One member of the research team will contact patients by telephone within 24 hours of the consultation with the GP in order to give explanations regarding the appropriate form of filling in the assessment booklet. At this time, other baseline outcomes will be recorded and then the patient will be randomised to study groups. The patient will be advised to keep the booklet at home, to seal it into the pre-paid envelope after the final assessment and mail the sealed envelope to the research team. To ensure the proper use of the assessment booklet and to avoid loss of data due to non-returned booklets, a blinded assessor will contact all patients by telephone 9 and 22 days after the consultation with the GP to collect patient's answers from the 1^st ^week and 3^rd ^week assessments, respectively.

The procedure for obtaining outcome data will be followed for all participants, regardless of compliance with trial protocols. At 3 months, data regarding the presence of persistent (chronic) symptoms will be collected by telephone. Participants will be asked to answer the following yes-no question: "During the past 3 months have you ever been completely free of low back pain? By this I mean no low back pain at all and would this pain-free period have lasted for a whole month". Those answering no will be considered to have persistent LBP. Information on additional treatment and the direct costs with low back pain management will also be collected at 3 months.

A secondary analysis will be performed on predictors of response to McKenzie treatment and prediction of chronicity. This will involve the measurement of participants' expectation about the helpfulness of both treatments under investigation as well as information on the occurrence of the centralisation phenomenon. Expectation will be recorded prior to randomisation according to the procedures described by Kalauokalani et al[38].

### Treatments

All participants will receive GP care as advocated by the NHMRC guideline for the management of acute musculoskeletal pain[2]. Guideline-based GP care consists of providing information on a favourable prognosis of acute LBP and advising patients to stay active, together with the prescription of paracetamol. Patients randomised to the experimental group will be referred to physiotherapy to receive the McKenzie Method. A research assistant not involved in the assessment or treatment of subjects will be responsible for the randomisation process and will contact therapists and patients to arrange the first physiotherapy session. The McKenzie treatment will be delivered by credentialed physiotherapists who will follow the treatment principles described in McKenzie's text book[18]. All therapists will have completed the four basic courses taught by the McKenzie Institute International. To ensure the appropriate implementation of the McKenzie's classification algorithm, a training session with a member of McKenzie's educational program will be conducted prior to the commencement of the study. The treatment frequency will be at the discretion of the therapist with a maximum of 7 sessions over 3 weeks. We chose to restrict the McKenzie treatment to a maximum of 7 sessions based on the study of Werneke and colleagues[39], which concluded that further reductions in pain and function are not expected if favourable changes in pain location are not present until the seventh treatment visit. Treatment procedures from the McKenzie Method are summarised in the Appendix.

Participants randomised to the control group will continue their GP care as usual. All participants regardless of intervention group will be advised not to seek other treatments for their low back pain during the treatment period. Physiotherapists will be asked to withhold co-interventions during the course of the trial.

Several mechanisms will be used to ensure that the trial protocol is applied consistently. Protocol manuals will be developed and all involved researchers (GPs, physiotherapists, assessor, and statistician) will be trained to ensure that screening, assessment, random allocation and treatment procedures are conducted according to the protocol. A random sample of treatment sessions will be audited to check that treatment is being administered according to the protocol.

### Data analysis

Power was calculated based on the primary outcome measures (pain intensity and global perceived effect). A sample size of 148 participants will provide 80% power to detect a difference of 1 unit (15%) on a 0–10 pain scale (SD = 2.0) between the experimental and control groups, assuming alpha of 0.05. This allows for loss to follow-up of 15%. This sample size also allows the detection of a difference of 1.2 units (12%) on a 0–10 global perceived effect scale (SD = 2.4).

Data will be analysed by a research member blinded to group status. The primary analysis will be by intention-to-treat. In order to estimate treatment effects, between-group mean differences (95%CI) will be calculated for all outcome measures. In the primary analysis these will be calculated using linear models that include baseline values of outcome variables as covariates to maximise precision.

## Discussion

We have presented the rationale and design of an RCT evaluating the effects of the McKenzie Method in the treatment of acute non-specific LBP. The results of this trial will be presented as soon as they are available.

## Competing interests

The author(s) declare that they have no competing interests.

## Authors' contributions

LACM, CGM and RDH were responsible for the design of the study. HC was responsible for recruiting McKenzie therapists and she will also participate as a clinician in the trial. LACM and JMc will act as trial coordinators. All authors have read and approved the final manuscript.

## Appendix

Clinical picture and treatment principles according to the McKenzie Method

This table summarises the procedures involved in the McKenzie Method (Table 1).  For detailed description of all procedures and progressions, refer to McKenzie's text book. This is particularly important for Derangement syndrome since the treatment is extremely variable and complex and the full description of procedures would not be appropriate for the purposes of this paper.

**Table 1:** 

***Postural Syndrome***	
**Clinical picture**	Intermittent back pain under prolonged, static end-range postures (usually flexion); no loss of movement, absence of deformity.
**Treatment**	Patient education and postural correction.
**Procedures**	Patient adopts the posture that produces their symptoms. Physiotherapist instructs patient how to abolish symptoms by correcting the posture and provides explanation on the mechanism that produces pain of postural origin. Attainment of the corrected posture is taught through the use of the "slouch-overcorrect" exercise. Patients are taught how to maintain the corrected posture through the use of a Lumbar roll and actively when a lumbar roll can-not be used. Consequences of postural neglect are discussed.
***Dysfunction Syndrome***	
**Clinical picture**	Intermittent back pain at premature end-range; radiation only in the case of the dysfunction of an adherent nerve root; partial loss of movement.
**Treatment**	Patient education, postural correction, and stretching of contracted structures.
**Procedures**	Posture correction and repeated end-range movements towards the direction of dysfunction (e.g. extension exercises for extension dysfunction). Ten to 15 stretches are repeated at 2/3-hourly intervals, until the movement loss is restored. Treatment progression may include clinician overpressure and/or mobilisation.
***Derangement Syndrome***	
**Clinical picture**	Constant or intermittent back pain and/or leg pain that moves proximally or distally during repeated movements; variable degree of loss of movement; deformity, paraesthesia, numbness and myotomal weakness may be present. A rapid change in the location of symptoms and in the range of movement is seen.
**Treatment**	Reduction of derangement and maintenance of reduction, recovery of function and prophylaxis.
**Procedures**	Reduction of derangement is achieved with sustained positions and/or repeated end-range movements. The treatment principle (extension, flexion or lateral) is selected according to the movements that abolish, decrease or centralise symptoms, as well as those that restore mobility and function (e.g. extension principle is adopted when extension centralises symptoms). Patient generated forces are used as the procedure of first choice. The exercises are repeated at home at 2-hourly intervals or as necessary for pain relief. Forces are progressed when the progress plateaus including over-pressures and therapist mobilisation. To ensure the maintenance of the reduction the patient is instructed to avoid aggravating postures or movements. Lumbar supports are used where necessary for the maintenance of lumbar lordosis.

## Pre-publication history

The pre-publication history for this paper can be accessed here:


